# Monocyte Based Correlates of Immune Activation and Viremia in HIV-Infected Long-Term Non-Progressors

**DOI:** 10.3389/fimmu.2019.02849

**Published:** 2019-12-06

**Authors:** Varsha M. Prabhu, Amit Kumar Singh, Varsha Padwal, Vidya Nagar, Priya Patil, Vainav Patel

**Affiliations:** ^1^Department of Biochemistry and Virology, National Institute for Research in Reproductive Health, Indian Council of Medical Research, Mumbai, India; ^2^Department of Medicine, The Grant Medical College and Sir J. J. Group of Hospitals, Mumbai, India

**Keywords:** monocyte, HIV-1, LTNP, immune activation, CD206, viremia, SNAE

## Abstract

**Background:** Disease progression monitoring through CD4 counts alone can be inadequate in HIV infection as ongoing immune activation may result in Serious non-AIDS events (SNAEs). SNAEs involve monocyte activation driven chronic inflammation with significant sequelae observed even during HAART. Here, we attempted to delineate functional monocyte based signatures across stages of HIV disease progression.

**Methods:** Participants spanning four cohorts were recruited—pre-ART (PA; <7 years of infection; *n* = 20), long-term non-progressors (LTNP; >7 years of infection, CD4 > 350 cells/μL, *n* = 20), individuals on therapy (ART; *n* = 18) and seronegative controls (SN; *n* = 15). Immunophenotyping of monocyte subsets and evaluation of expression of HIV-binding receptors—CD4 and CCR5, marker of immune activation- HLA-DR and M2 phenotype—mannose receptor (CD206) was followed by association of monocyte-specific parameters with conventional markers of disease progression such as absolute CD4 count, CD4/CD8 ratio, viral load, and T cell activation.

**Results:** A significant expansion of intermediate monocytes (CD14++CD16+) with a concomitant decline in classical subset (CD14++CD16–) was observed in all infected cohorts compared to seronegative controls. In addition, an expansion of the non-classical subset (CD14+CD16++) was observed in long-term non-progressors. Dysregulation in monocyte subsets associated with CD4 count and CD4/CD8 ratio in PAs but not in LTNPs. We report for the first time that expression of CD206 is most prominent on intermediate monocytes which also have the highest expression of CD4, CCR5, and HLA-DR. Despite preserved CD4 counts, LTNPs had similar immune activation profiles to PAs, as evidenced by elevated HLA-DR expression across monocyte subsets. HLA-DR expression, similar to that in SNs, observed in the ART group indicated partial immune restoration within the monocyte compartment. Increased CD206 expression on monocytes together with frequency of activated CD4+ T lymphocytes (HLA-DR+CD38+) showed significant and positive association with viral load in LTNPs, but not PAs.

**Conclusion:** Our results describe for the first time the presence of monocyte dysregulation involving increased activation in LTNPs, who, in spite of preserved CD4 counts, may remain susceptible to prolonged effects of systemic inflammation and highlight CD206, as a unique non-T correlate of viremia, in viremic non-progression.

## Introduction

Monocytes are a heterogeneous leukocyte population that can be delineated based on their expression of CD14 and CD16 into three distinct subsets. In healthy individuals, classical monocytes (CD14++CD16–), the predominant population, comprise ~85% of all circulating monocytes. CD16 expressing monocytes, more recently reported on, constitute ~10% of the whole and numerous studies have highlighted a developmental hierarchy from classical through intermediate (CD14++CD16+) to non-classical (CD14+CD16++) monocytes (1–3). CD16+ monocytes represent a more mature and activated phenotype that expands in various pathologies including HIV infection (1, 4–6).

Serious non-AIDS events (SNAEs) encompass a gamut of conditions such as cardiovascular disease (CVD), neurological impairment, non-AIDS malignancies, renal, hepatic and bone disorders associated with chronic HIV infection (7). Monocyte activation, manifested as increased frequencies of intermediate and non-classical monocytes and upregulation of soluble markers of inflammation such as neopterin in HIV infected individuals, is a key factor in the development of CVD (8–10). Microbial translocation, a driver of systemic immune activation, also induces CD16+ monocyte expansion and transmigration across the blood-brain barrier contributing to HIV-associated neurocognitive disorders (HAND) (11, 12).

The current paradigm for initiation of antiretroviral therapy (ART) is that of test-and-treat to enable better HIV transmission outcomes and facilitate immune restoration (13, 14). While effective therapy can achieve viral suppression and CD4 rebound, levels of immune activation markers do not fully normalize despite ART (15–17). Thus, persistent immune activation due to residual viremia, microbial translocation, co-infections and altered homeostasis contribute to development of SNAEs in ART-mediated delayed progression (7).

Long-term non-progressors, a unique group of HIV-infected individuals, maintain stable CD4 counts (350–1,600 cells/μL) in the absence of therapy for extended duration. Based on viremia, they may be categorized as elite controllers with <50 copies/mL, viremic controllers (50–2,000 copies/mL) and a rare subset of viremic non-progressors (VNPs) with sustained CD4 counts despite moderate to high viral replication (18, 19). In a seminal study, elite controllers displayed higher median frequencies of activated CD8+ T cells compared to seronegative controls and treated participants with undetectable viremia (20). Viremic non-progressors also had similar levels of T cell activation as putative progressors, suggesting that preservation of CD4 compartment does not preclude non-progressors from the deleterious effects of chronic T cell activation (21).

While immune activation is a well-documented feature both in chronic HIV infection as well as in elite controllers, relatively fewer studies have reported on non-progressors with or without viremia and have predominantly addressed T cell activation. Evaluating monocyte dysregulation and activation as risk factors for the development of SNAEs, in the context of non-progression, would provide valuable and heretofore unavailable non-T cell correlates of HIV pathogenesis. Towards this end, we examined functionally-distinct monocyte subsets across a spectrum of disease progression states in HIV infection.

## Materials and Methods

### Study Participants

All samples were collected from individuals attending the ART center at Sir. J. J. Group of Hospitals, Mumbai with informed consent from the participants and approval from the ICMR- NIRRH Ethics Committee for Clinical Studies (Project No. **225/2012**). Enrolled participants spanned four cohorts—HIV-seronegative (SN, *n* = 15), pre-ART (PA, *n* = 20), long-term non-progressors (LTNP, *n* = 20), and individuals on antiretroviral therapy (ART, *n* = 18). Long-term non-progressors were defined as individuals maintaining stable CD4 counts >350 cells/μL for at least 7 years after initial detection of HIV infection (22). Viral nucleic acid was isolated from blood plasma using the MagNA Pure Compact Instrument with their Nucleic Acid Isolation kit (Roche Diagnostic, Germany) and plasma viral load was estimated by COBAS TaqMan 48 Analyzer using the COBAS^®^ TaqMan^®^HIV-1 Test kit (Roche) with 34 copies/mL being the limit of detection. The clinical characteristics of participants such as age, gender, duration of infection, absolute CD4 count, viral load, and ART status are summarized in Table 1.

**Table 1:** Clinical characteristics of participants.

	**Seronegative (*n* = 15)**	**Pre-ART(PA) (*n* = 20)**	**LTNP (*n* = 20)**	**ART (*n* = 18)**
Age^α^, years	35 (22–50)	39 (23–55)	40 (12–60)	43.5 (15–51)
Male/female (M/F)	11M/4F	10M/10F	4M/16F	12M/6F
Absolute CD4 count^α^, cells/μL	876.5 (527–1254)	528 (197–877)	636.1 (407–1253)	622 (184–1235)
Viral load^α^, log (copies/mL)	–	4.62 (3.18–6.09)	4.40 (2.95–5.85)	UD^‡^ (8),2.42 (8) (1.71–3.58)°
Duration of infection^α^, ^~^, years	–	1 (0–6)	10 (7–18)	7.9 (2.2–20)
Duration on ART^α^, years	–	–	–	3.96 (1–10.25)
ART regimen	–	–	–	ALE (1), ALN (3), ZLN (5), TLE (2), TL-ATV (6)^*^
CD4 recovery post-ART^α^ (fold-change)	–	–	–	4.897^∧^ (1.36–13.62)

α *Data expressed as median followed by range*.

‡ *Undetectable (<34 copies/mL)*.

*°Viral load data is from 16 individuals on ART, eight had viral loads below the limit of detection and data was unavailable for two*.

~ *Duration of infection was estimated from date of diagnosis*.

* *ALE, Azidothymidine Lamivudine Efavirenz; ALN, Nevirapine; ZLN, Zidovudine; TLE, Tenofovir; TL- ATV (Second line regimen); ATV, Atazanavir*.

∧ *Data from 14 individuals where pre-ART CD4 counts were known*.

### Immunophenotyping and Flow Cytometry

Monocytes were identified based on forward and side scatter properties and expression of CD14 and CD16. The expression of dim markers was confirmed based on fluorescence −1 (FMO) controls. Briefly, peripheral blood was collected in EDTA vacutainers and 200 μL was incubated with fluorochrome conjugated antibodies specific for cell-surface markers on monocyte subsets. Anti-CD14 (Clone: M5E2), anti-CD16 (Clone: 3G8), anti-CD4 (Clone: RPA-T4), anti-CCR5 (Clone: 2D7), anti-HLADR (Clone: L243), and anti-CD206 (Clone: 15-2). All antibodies were purchased from either BD Biosciences or BioLegend (US). For chemokine receptor CCR5, staining was carried out at 37°C for 15 min followed by incubation with remaining antibodies (CD14/CD16/CD4/HLADR) at RT for 15 min as described previously (23). A similar protocol was performed for CD206 which undergoes endocytic recycling, whereby staining at physiological temperatures would allow receptor cycling to the surface and maximal binding of antibody. Stained samples were incubated with ice-cold FACS Lyse buffer (BD Biosciences) for 15 min with intermittent vortexing to ensure complete RBC lysis and washed twice with staining buffer (PBS with 0.2% FBS). *Ex-vivo* staining was carried out within 3 h of sample collection and roughly 30,000 events were acquired within a monocyte gate on the BD Accuri C6 Flow Cytometer (BD Biosciences). Data analysis was carried out on FlowJo 10.2 (Tree Star Inc., Ashland, Oregon, USA).

T cell activation was estimated using anti-CD3 (Clone: SK7), anti-CD8 (Clone: SK1), anti-CD38 (Clone: HIT2), and anti-HLADR (Clone: L243) and examining the frequency of HLADR/CD38 dual-positive cells within CD4+ and CD8+ T lymphocyte gates as described previously (24–26). The frequency of regulatory T cells was estimated using anti-CD3 (Clone: SK7), anti-CD4 (Clone: RPA-T4), anti-CD25 (Clone: M-A251), and anti-CD127 (Clone: HIL-7R-M21) as described previously (26).

### Statistical Analysis

All statistical analyses were performed using GraphPad Prism 6.01 (GraphPad Software, San Diego, California, USA). Data has been represented as scatter plots with bars indicating median values. Comparison between groups was made using Kruskal-Wallis ANOVA with *post hoc* Dunn's multiple comparisons test and unpaired *t*-test with Welch's correction at 95% confidence interval. Association between variables was assessed using linear regression and non-parametric Spearman correlation analyses.

## Results

### Participant Characteristics

As summarized in Table 1 and shown in Supplementary Figure 1B, all infected cohorts had lower median CD4 counts than seronegative control group (SN, median CD4 = 876.5 cells/μL). LTNPs, as expected, had higher absolute CD4 counts (median−636.1 cells/μL) than PA (median−528 cells/μL). Individuals on therapy displayed a range of CD4 counts (ART, median−622 cells/μL) which reflected partial rebound in all individuals (Supplementary Figure 1F). The ART-naïve groups had comparable viral loads (PA, median−4.62 logs and LTNP, median−4.40 logs), indicating the presence of viremic non-progressors (VNPs) within the LTNP cohort. The LTNP cohort had a median duration of 10 (7–18) years since HIV diagnosis. The ART group comprised of individuals on therapy for at least 1 year, with undetectable viremia (*n* = 8) or <1,000 copies/mL (*n* = 7) and included six individuals receiving the second line regimen. One individual in this group, receiving 2nd line therapy (TL-ATV) had viremia above the WHO criteria of failure (3,887 copies/ml) but showed a significant rebound of CD4 count (144–1,049 cells/μL) at the time of sampling. All groups were age-matched and did not show any significant difference (Kruskal-Wallis *H* = 3.307, *P* = 0.3467) in median age compared to seronegative controls (Supplementary Figure 1A). The groups were not sex-matched and the LTNP group in our study was enriched for female participants as observed previously (18). The clinical characteristics of recruited participants have been graphically represented in Supplementary Figures 1A–F.

### Dysregulation in Frequencies of Monocyte Subsets Across All Infected Cohorts

**Figure 1:**
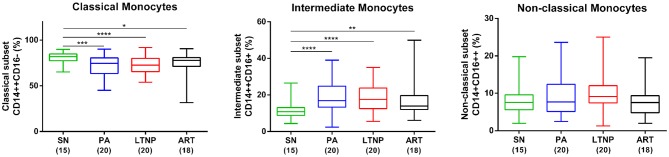
Altered frequencies of monocyte subsets in HIV infection. Box and whisker plots showing comparison of frequency of monocyte subsets in PA (*n* = 20), LTNP (*n* = 20), and ART (*n* = 18) groups with seronegative controls (SN, *n* = 15) (horizontal bar indicates median). Statistical significance was estimated by Kruskal-Wallis ANOVA followed by Dunn's multiple comparison test; ^*^P < 0.05; ^**^P < 0.01; ^***^P < 0.001; ^****^P < 0.0001. The expansion of non-classical subset (right-most panel) in ART-naïve groups was not significant by ANOVA but was observed to be significantly higher in LTNPs compared to both SN and ART groups by unpaired *t*-test (*P* < 0.05 for both).

To begin with, we examined the frequency of monocyte subsets in whole blood across different states of disease progression. The gating strategy used to differentiate between classical (CD14++CD16−), intermediate (CD14++CD16+) and non-classical (CD14+CD16++) monocytes as per previously established nomenclature (27) is shown in Supplementary Figure 2 with representative plots for each cohort. As shown in Figure 1, we observed a significant decline in the median frequency of classical monocytes with a concomitant expansion of the intermediate subset in all infected cohorts compared to seronegative controls [SN, classical subset, median = 81.85%, interquartile range (IQR) = 77.35–84.83%; intermediate subset, median = 10.80%, IQR = 8.84–13.10%)]. Median frequencies of these monocyte subsets in therapy-naïve (PA, classical, median = 74.50%, IQR = 63.60–80.50%; intermediate, median = 16.90%, IQR = 13.30–24.80%) and treated individuals (ART, classical, median = 77.85%, IQR = 71.23–80.83%; intermediate, median = 13.95%, IQR = 12.08–19.65%) did not differ significantly suggesting that extended ART was ineffective in restoring this imbalance (Figure 1). Interestingly, the perturbation in monocyte subsets was present in non-progressors as well (LTNP, classical, median = 72.65%, IQR = 65.50–80.08%; intermediate, median = 17.50%, IQR = 12.55–23.78%). A modest rise in the frequency of non-classical monocytes in both ART-naïve groups (PA, median = 7.68%, IQR = 5.13–12.40%) including non-progression (LTNP, median = 9.14%, IQR = 7.45–12.05%) did not reach significance compared to seronegative controls (SN, median = 7.56%, IQR = 5.62–9.58%) by one-way ANOVA. However, it was observed to be significantly higher in LTNPs compared to both SN and ART groups by pairwise comparisons, respectively (*P* < 0.05 for both). Expansion of non-classical subset (ART, median = 7.53%, IQR = 4.82–9.36%) was not observed, compared to seronegative controls in the ART group, indicative of partial immune restoration.

### Association of Monocyte Subsets With Markers of Disease Progression

**Figure 2:**
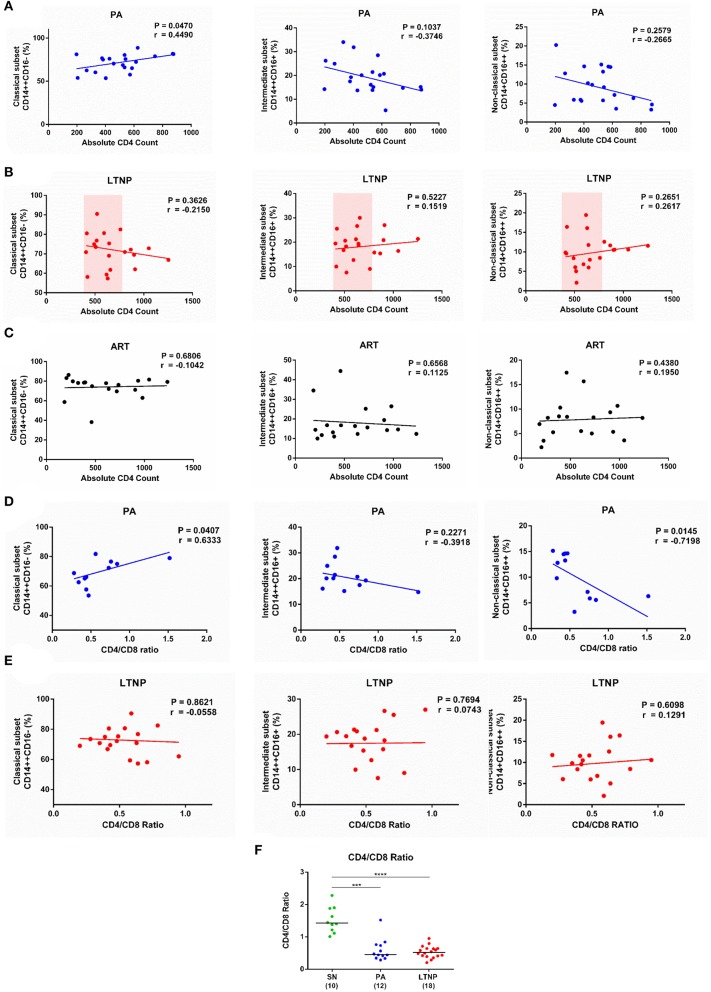
Association of monocyte subsets with CD4 count and CD4/CD8 Ratio. **(A–C)** Correlation analyses of monocyte subset frequencies with absolute CD4 count in the PA (*n* = 20), LTNP (*n* = 20), and ART (*n* = 18) groups, respectively. Note the clustering of absolute CD4 counts around the median value of 500 cells/μL in the LTNP group. **(D,E)** Correlation analyses of frequency of classical, intermediate and non-classical monocytes with CD4/CD8 ratio in PA (*n* = 12) and LTNP (*n* = 18) groups, respectively. Association between variables was evaluated by linear regression and Spearman correlation test was used to determine correlation co-efficient (*r*) and significance (*P* < 0.05). **(F)** Comparison of CD4/CD8 ratios of therapy-naïve groups (PA and LTNP) with seronegative (SN) controls. Statistical significance was estimated by Kruskal-Wallis ANOVA followed by Dunn's multiple comparison test; ^***^P < 0.001; ^****^P < 0.0001.

In order to understand whether the apparent imbalance in frequency of monocyte sub-populations is associated with conventional markers of disease progression we correlated frequency of monocyte subsets with absolute CD4 count in each cohort. In the PA group, as previously reported (8, 28), the frequency of classical monocytes showed a strong positive correlation with absolute CD4 count (*r* = 0.45, *P* < 0.05). An inverse association observed in both CD16 expressing monocyte subsets, intermediate (*r* = −0.37, *P* = 0.10) and non-classical (*r* = −0.27, *P* = 0.26), did not reach significance (Figure 2A). In the ART and LTNP groups however, no such association was observed, possibly due to altered homeostasis of the CD4 compartment by therapy (Figure 2C) and clustering of CD4 counts around the median value of 500 cells/μL (Figure 2B), respectively. For the PA and LTNP groups, we were also able to examine CD4/CD8 ratio, a more robust marker of disease progression, which has been associated with increased immune activation and higher risk of developing SNAEs (29). In the PA group a trend emerged that was similar to that observed with absolute CD4 counts and the association with CD4/CD8 ratio was found to be significant in classical (*r* = 0.63, *P* < 0.05) and non-classical (*r* = −0.72, *P* < 0.05) subsets (Figure 2D). Interestingly however, no significant association of monocyte subsets with CD4/CD8 ratio was observed in the LTNP group (median CD4/CD8 ratio = 0.51, IQR = 0.40–0.64) (Figure 2E) despite having a wide range of CD4/CD8 ratios whose distribution was similar to the PA group (median = 0.45, IQR = 0.36–0.75), but still lower than SN individuals (median = 1.42, IQR = 1.18–1.88) (Figure 2F). Thus, altered frequencies of monocyte subsets while observed in all infected groups may reflect disparate immune homeostatic mechanisms unique to particular states of disease progression.

### HIV-Binding Receptors in LTNPs and Progressors

**Figure 3:**
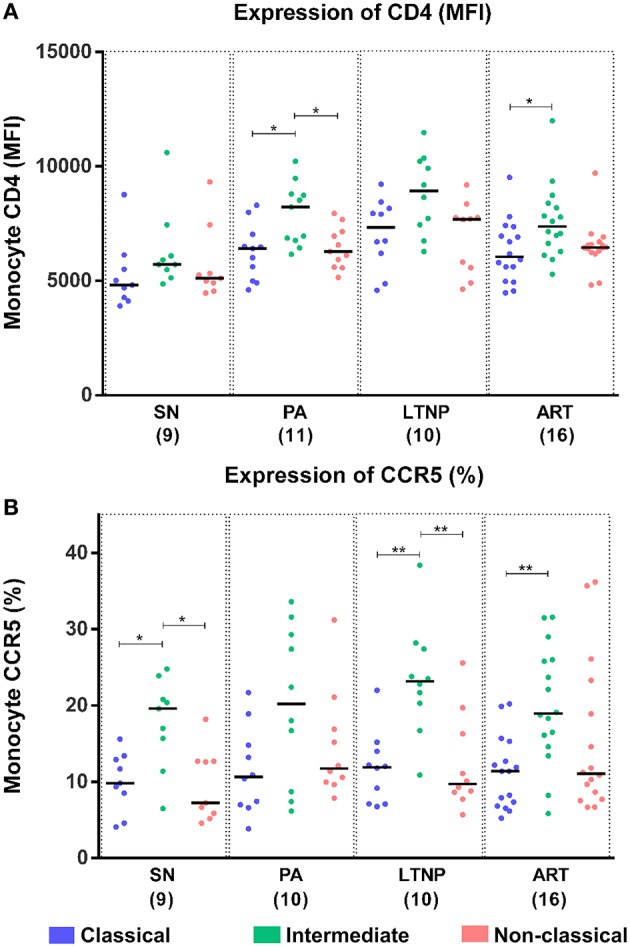
Differential expression of HIV-binding receptors on monocyte subsets. **(A)** Per-cell expression of HIV-binding receptor CD4 (MFI) was highest on the intermediate monocyte subset across cohorts. Kruskal-Wallis ANOVA followed by Dunn's multiple comparison test was used to assess significance. Although not significant by ANOVA, per-cell expression of CD4 was observed to be significantly higher on intermediate monocytes in the LTNP group using unpaired *t*-test (*P* < 0.05 for both classical and non-classical subsets). **(B)** Expression of HIV co-receptor CCR5 (% positivity) was highest on intermediate monocytes across different disease progression states. The plot reflects results of Kruskal-Wallis ANOVA followed by Dunn's multiple comparison test to assess significance; ^*^P < 0.05; ^**^P < 0.01.

Although relatively refractory to infection, monocytes are known to harbor replication-competent HIV and serve as potential reservoirs (30). We sought to examine the expression of canonical HIV binding receptor CD4 and co-receptor CCR5 on different monocyte subsets as a potential contributing factor to varying disease states including non-progressive infection that was accompanied by viremia. The per cell expression of CD4 (MFI) was significantly higher on intermediate monocytes compared to both classical and non-classical subsets in the PA group and compared to the classical subset alone in the ART group. The apparent increase in per-cell expression of CD4 on intermediate monocytes did not reach significance for the LTNP group by ANOVA but was significant by pairwise comparisons (*P* < 0.05 for both classical and non-classical) (Figure 3A). This differential expression was most pronounced in the ART-naïve groups where per-cell expression of CD4 was 23.05% [13.79–37.03%] and 26.40% [IQR = 20.3–33.44%] (*P* < 0.05) higher on intermediate monocytes than non-classical monocytes in PAs and LTNPs, respectively, compared to the 11.79% [IQR = 3.68–14.32%] increment observed in SN controls (Supplementary Figure 3B, right panel). On examining the expression of CCR5 across monocyte subsets, we observed that the intermediate subset showed both higher frequency of CCR5+ cells and greater per-cell expression as compared to classical and non-classical subsets in all cohorts (Figure 3B, Supplementary Figure 3A). However, the fold-change in frequency of CCR5 positive cells between intermediate and non-classical subsets varied across groups with non-progressors [median = 2.35, IQR = 1.16–2.83] displaying a pattern of expression similar to SN individuals [median = 1.95, IQR = 1.18–2.8], whereas PAs [median = 1.275, IQR = 0.87–2.13] closely resembled individuals on therapy [median = 1.58, IQR = 0.83–2.35] (Supplementary Figures 3C,D). Our observations are the first to compare CD4 and CCR5 expression in monocyte subsets for LTNP and PA individuals. They delineate a monocyte CD4 expression pattern for PA and LTNP individuals where variance in per cell expression across subsets is higher in these groups compared to therapy-receiving individuals. Conversely, frequency distribution of CCR5+ cells in LTNPs aligned with SN controls, not PAs.

### Association of Monocyte Activation With M2 (Alternate Activation) Marker CD206

**Figure 4:**
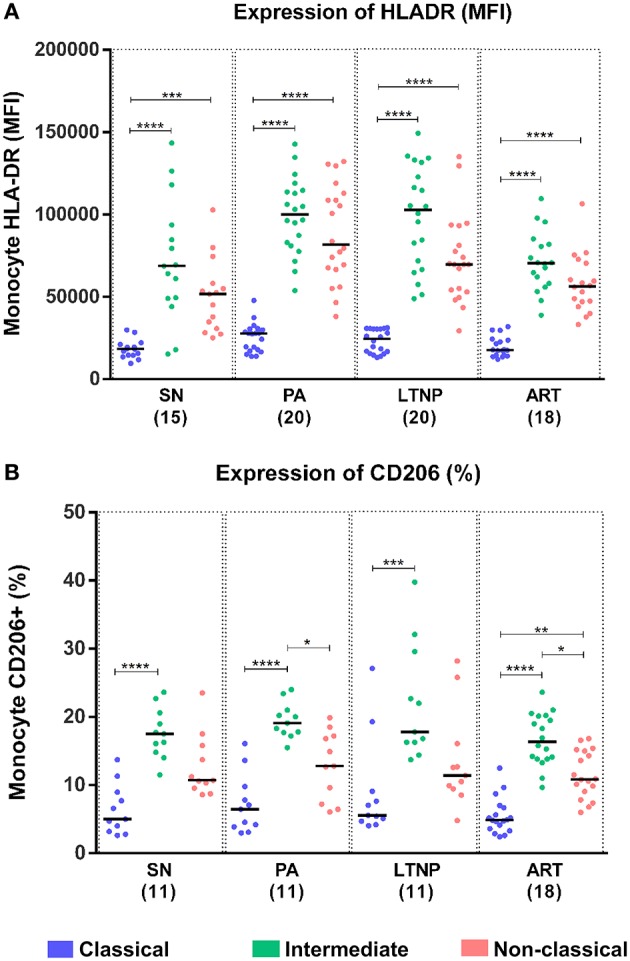
Differential expression of activation markers on monocytes subsets. **(A,B)** Expression of monocyte activation marker—HLA-DR (MFI) and marker for M2 phenotype—CD206 (%), respectively across monocyte subsets for each cohort. Kruskal-Wallis ANOVA followed by Dunn's multiple comparison test was used to assess significance; ^*^P < 0.05; ^**^P < 0.01; ^***^P < 0.001; ^****^P < 0.0001.

Monocytes constitutively express HLA-DR, a known marker of activation that is upregulated in HIV infection and is most expressed on the intermediate subset (4, 31, 32). However, no data on HLA-DR expression in monocyte subsets for LTNP individuals has been reported. We found per-cell expression of HLA-DR on intermediate monocytes to be ~4-fold higher as compared to that on classical monocytes (*P* < 0.001 for all) in all cohorts (Figure 4A). HLA-DR expression was also ~3-fold higher on non-classical monocytes as compared to classical monocytes across cohorts (SN, *P* < 0.001; *P* < 0.0001 for rest) (Figure 4A). Furthermore, we investigated the expression of CD206 across monocyte subsets as a recently reported marker of alternate-activation (M2) in these cells (33–35). We report for the first time that CD206 is expressed highest on the intermediate subset followed by non-classical subset in seronegative controls (SN) and all infected cohorts in terms of percentage positivity and median fluorescence intensity (Figure 4B, Supplementary Figures 5A, 7A). Intermediate monocytes express ~3–3.5-fold higher levels of CD206 than classical monocytes (SN, *P* < 0.0001; PA, *P* < 0.0001; LTNP, *P* < 0.001; ART, *P* < 0.0001), and ~1.5-fold higher levels than non-classical monocytes (PA, *P* < 0.05; ART, *P* < 0.05) (Figure 4B). The differential expression of HLA-DR and CD206 across subsets for each cohort is shown in Supplementary Figures 4A,B, 5B, respectively. Interestingly, we observed a significant association between per-cell expression of HLA-DR and CD206 positivity on intermediate monocytes in seronegative controls (*r* = 0.6848, *P* < 0.05) and treated individuals (ART, *r* = 0.5382, *P* < 0.05), that was absent in both ART-naïve groups (PA: *r* = 0.2909, *P* = 0.38 and LTNP: *r* = −0.1412, *P* = 0.66) (Table 2).

**Table 2:** Association of CD206 with HLA-DR expression on monocytes subsets.

**Group**	***N***	**Classical**	**Intermediate**	**Non-classical**
SN	11	*P* = 0.6567 *r* = 0.1636	***P*** **=** **0.0347** ***r*** **=** **0.6848**	*P* = 0.9460 *r* = −0.0303
PA	11	*P* = 0.9462 *r* = −0.0273	*P* = 0.3863 *r* = 0.2909	*P* = 0.0609 *r* = 0.5909
LTNP	11	*P* = 0.8870 *r* = −0.0456	*P* = 0.6673 *r* = −0.1412	*P* = 0.7530 *r* = 0.1101
ART	18	*P* = 0.4353 *r* = 0.1962	***P*** **=** **0.0212** ***r*** **=** **0.5382**	*P* = 0.0776 *r* = 0.4264

*Bold font if P value is significant*.

### Altered Expression of HIV-Binding Receptors Across Cohorts

**Figure 5:**
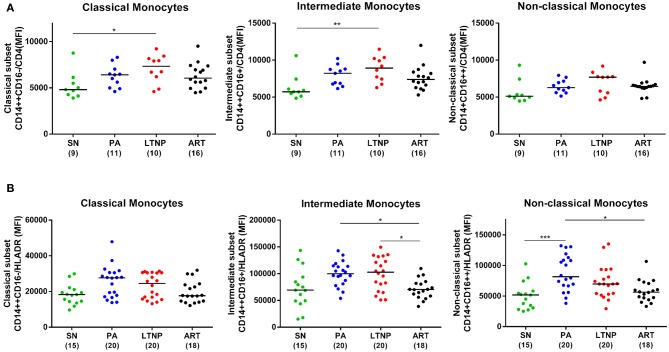
Expression of CD4 and HLA-DR on monocyte subsets across cohorts. **(A)** Comparison of CD4 per-cell expression across PA, LTNP, ART and seronegative (SN) control groups in different monocyte subsets. Significance was ascertained using Kruskal-Wallis ANOVA followed by Dunn's multiple comparison test; ^*^P < 0.05; ^**^P < 0.01; ^***^P < 0.001. Elevated CD4 expression on intermediate monocytes was also observed to be significantly higher in PAs than SN individuals by unpaired *t*-test (*P* < 0.05). **(B)** Comparison of per-cell expression of HLA-DR across cohorts in monocyte subsets. The plots reflect results of Kruskal-Wallis ANOVA followed by Dunn's multiple comparison test. In addition, both ART-naïve groups showed significantly higher HLA-DR expression compared to seronegative controls across monocyte subsets based on pairwise comparisons by unpaired *t*-test (Classical—PA, *P* < 0.01; LTNP, *P* < 0.05; intermediate—*P* < 0.05 for both; non-classical—PA, *P* < 0.001; LTNP, *P* < 0.05).

Subsequently, we compared the expression of CD4 on intermediate monocytes across cohorts and observed elevated levels in both ART-naïve groups that was significant in LTNPs (~40%, *P* < 0.01) compared to seronegative controls (SN) and was moderately suppressed by therapy in the ART group (~20%, *P* = 0.09) (Figure 5A). A similar trend was observed for CD4 expression on the classical subset and non-classical subsets but reached significance only for the LTNP group (~35%, *P* < 0.05) on classical monocytes compared to seronegative controls (Figure 5A).

There was no significant alteration in CCR5 expression across cohorts in terms of percentage positivity and median fluorescence intensity (Supplementary Figures 6A,B, respectively). We did observe an inverse association between expression of CCR5 (MFI) on classical (*r* = −0.5818, *P* = 0.06) and intermediate (*r* = −0.5727, *P* = 0.07) subsets with absolute CD4 counts in the PA group and a similar significant association of CCR5 with classical (*r* = −0.6284, *P* < 0.05) and intermediate (*r* = −0.6029, *P* < 0.05) subsets in the ART group (Supplementary Table 1). Such an association was absent in non-progressors (LTNP).

### Monocyte Activation in Therapy Naïve Disease

When we investigated HLA-DR expression subset-wise, across cohorts, we observed that it was elevated in both ART-naïve groups—PA and LTNP, irrespective of disease progression as compared to seronegative controls (Figure 5B). This observation was true for all three monocyte subsets (by pairwise comparison—unpaired *t*-test) suggesting an overall activation of monocytes in untreated infection. Thus, non-progressors, despite having preserved CD4 counts, showed signs of monocyte activation similar to putative progressors. Enhanced HLA-DR expression on the classical subset was significantly associated with viremia in the PA group (*r* = 0.4561, *P* = 0.05) and low CD4/CD8 ratios in the LTNP group (*r* = −0.4574, *P* = 0.05) (Supplementary Table 2). Interestingly, there was a significant association between HLA-DR expression on intermediate monocytes with elevated CD4 expression (Figure 5A) on the same subset in the LTNP group (*r* = 0.8061, *P* < 0.01) (Table 3). A similar and highly significant association was also observed between enhanced expression of HLA-DR and CD4 on the classical subset (*r* = 0.9483, *P* = 0.0001) within the LTNP group (Table 3).

**Table 3:** Association of CD4 and HLADR per-cell expression on monocyte subsets.

**Group**	***N***	**Classical**	**Intermediate**	**Non-classical**
SN	9	*P* = 0.2675 *r* = 0.4524	*P* = 0.2635 *r* = 0.5239	*P* = 0.9349 *r* = 0.04762
PA	12	*P* = 0.2336 *r* = 0.3909	*P* = 0.3560 *r* = 0.3091	*P* = 0.3269 *r* = 0.3273
LTNP	11	***P*** **=** **0.0001** ***r*** **=** **0.9483**	***P*** **=** **0.0072** ***r*** **=** **0.8061**	*P* = 0.3561 *r* = 0.3262
ART	16	*P* = 0.3183 *r* = −0.2636	***P*** **=** **0.0189** ***r*** **=** **0.5857**	*P* = 0.1963 *r* = −0.3387

*Bold font if P value is significant*.

### ART Mediated Suppression of Chronic Monocyte Activation

ART reduced the per cell expression of HLA-DR (MFI) on monocytes to the level of seronegative controls (Figure 5B), suggesting amelioration of chronic activation in the monocyte compartment through virological suppression. When compared to the PA group, individuals on therapy had significantly diminished HLA-DR expression-per cell (MFI) on intermediate (*P* < 0.05) and non-classical monocytes (*P* < 0.05). The ART group also displayed significantly reduced HLA-DR on the intermediate subset (*P* < 0.05) compared to LTNPs (Figure 5B). The decrease in HLA-DR expression on intermediate monocytes was significantly associated (*r* = 0.5857, *P* < 0.05) with reduced CD4 expression (MFI) on the same subset (Figure 5A) in the ART group similar to LTNPs (Table 3).

### CD206 Expression Is Elevated in Non-Progressors and Associated With Viral Load

**Figure 6:**
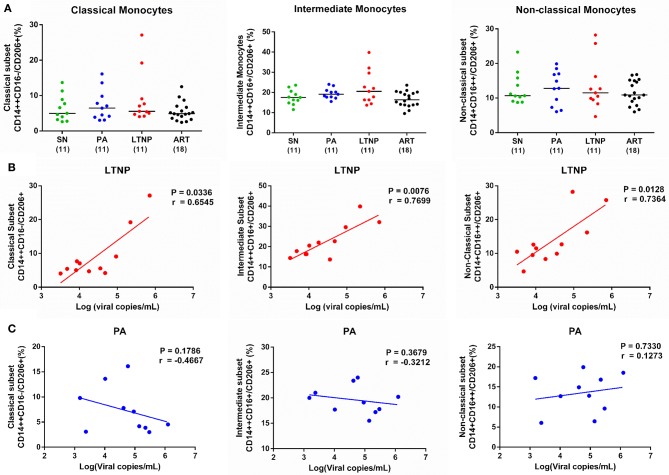
Expression of CD206 across cohorts and its association with viremia in therapy-naïve groups. **(A)** Expression of CD206 in monocyte subsets across PA (*n* = 11), LTNP (*n* = 11), ART (*n* = 18), and SN (*n* = 11) groups. Kruskal-Wallis ANOVA followed by Dunn's multiple comparison test was carried out to measure significance. The apparent increase in CD206 expression on intermediate monocytes was observed to be significantly higher in PA (*P* < 0.05) and LTNP (*P* = 0.05) groups compared to therapy receiving individuals using unpaired *t*-test. **(B,C)** Correlation analyses of CD206 on monocyte subsets with viremia in LTNP and PA groups, respectively. Association between variables is assessed using Spearman correlation analysis to determine correlation co-efficient “*r*” and test significance (*P* < 0.05).

Based on its association with HLA-DR, we sought to explore if CD206 expression too varies across different stages of progression. We observed an apparent increase in CD206 expression on intermediate monocytes in PA and LTNP groups as compared to individuals on therapy (ART) that was significant by pairwise comparisons using unpaired *t*-test (PA, *P* < 0.05; LTNP, *P* = 0.05) (Figure 6A). As the ART-naïve groups are characterized by uncontrolled viral replication we correlated CD206 expression with viral load in PA and LTNP groups. No association between CD206 and viremia was observed in the PA group across subsets (Figure 6C). Intriguingly, the association of CD206 on intermediate monocytes with viral load was highly significant (*r* = 0.7699, *P* < 0.01) in the LTNP group (Figure 6B). The correlation was also significant for CD206 expression on classical (*r* = 0.6545, *P* < 0.05) as well as non-classical (*r* = 0.7364, *p* < 0.05) monocytes in non-progressors (Figure 6B). In fact, despite the low numbers when stratified based on viral load, non-progressors with relatively high viremia (VL > 10,000 copies/mL) displayed significantly higher CD206 expression on intermediate monocytes than the ART group (*P* < 0.05), but not PAs (Supplementary Figures 7B,C).

### T Cell Activation and Monocyte Subsets in Therapy-Naïve Cohorts

**Figure 7:**
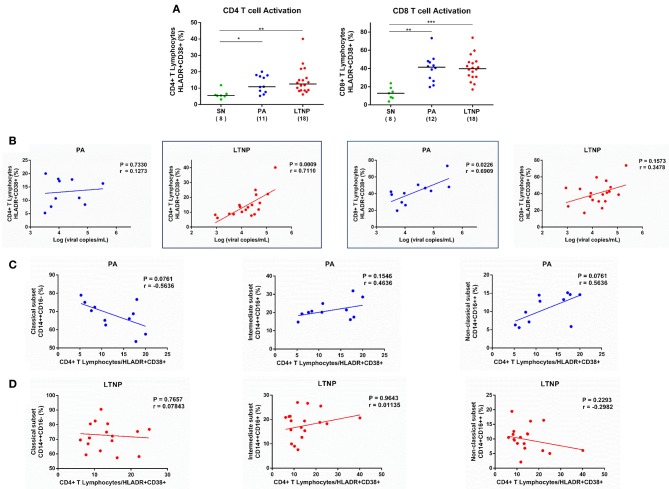
Association of T cell activation with viremia and frequency of monocyte subsets in therapy-naïve cohorts. **(A)** Comparison of CD4+ and CD8+ T lymphocyte activation in therapy-naïve groups (PA and LTNP) with seronegative (SN) controls. Kruskal-Wallis ANOVA followed by Dunn's multiple comparison test was applied to assess significance; ^*^P < 0.05; ^**^P < 0.01; ^***^P < 0.001. **(B)** Association of T cell activation with viremia in PA and LTNP groups. **(C,D)** Association of CD4+ T lymphocyte activation with frequency of monocyte subsets in PA and LTNP groups, respectively. Association between variables was assessed using Spearman correlation analysis to determine correlation co-efficient “*r*” and test significance (*P* < 0.05).

Chronic activation of the immune system is a hallmark of progressive HIV infection and frequency of activated T lymphocytes better predicts disease outcome than plasma viral load and CD4 counts alone (20, 26, 36). In our study, viral load showed a more significant association with frequency of activated CD8+ T lymphocytes (HLA-DR+CD38+) (*r* = 0.6909, *P* < 0.05) in the PA group (Figure 7B) and frequency of activated CD4+ T lymphocytes (HLA-DR+CD38+) (*r* = 0.7119, *P* < 0.001) in the LTNP group (Figure 7B). Furthermore, the frequency of classical monocytes showed an apparent negative association with CD4+ T cell activation and a positive association with non-classical monocytes in progressors (PA) (Figure 7C), while no such relationship was observed in non-progressors (LTNP) (Figure 7D). These discordant observations highlight subtle differences in immunopathology of cellular immune subsets in PA and LTNP groups having similarly higher levels of immune activation compared to seronegative controls (Figure 7A).

### Association of Regulatory T Cells With Monocyte Subsets in Therapy-Naïve Cohorts

**Figure 8:**
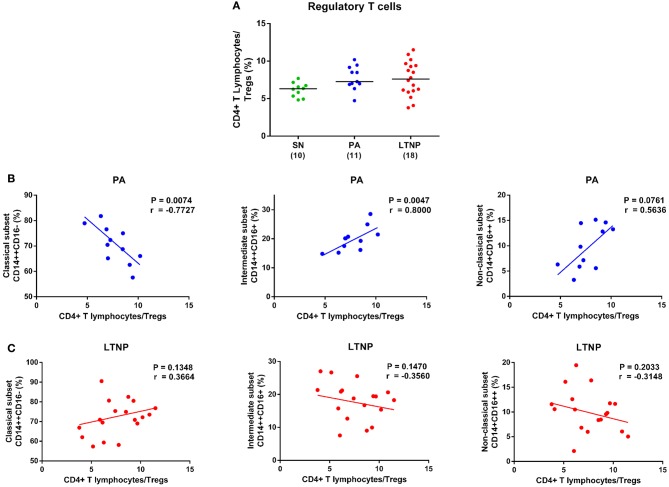
Association of regulatory T cells with monocyte subsets in therapy-naïve cohorts. **(A)** Comparison of regulatory T cell (Treg) frequency in therapy-naïve groups (PA and LTNP) with seronegative (SN) controls. Kruskal-Wallis ANOVA followed by Dunn's multiple comparison test was applied to assess significance. The expansion of regulatory T cell subset in both ART-naïve groups (PA and LTNP) compared to SN controls was significantly higher (*P* < 0.05 for both) based on pairwise comparisons using unpaired *t*-test. **(B,C)** Association of Treg frequency with frequency of monocyte subsets in PA and LTNP groups, respectively. Association between variables was assessed using Spearman correlation analysis to determine correlation co-efficient “*r*” and test significance (*P* < 0.05).

The expansion of regulatory T cell subset (Tregs) in HIV infection may serve a dual role by suppressing generalized immune activation and conversely mitigating HIV-specific immune responses (37). We observed anapparent increase in the frequency of Tregs (CD4+ CD25^high^ CD127^low^) in both PA and LTNP groups compared to SN controls (Figure 8A). This is in concurrence with our earlier reported data for both HIV-1 and HIV-2 infected individuals (16, 26). Expansion of Tregs exhibited a highly significant association with decline in the frequency of classical monocytes (*r* = −0.7727, *P* < 0.01) and corresponding rise in the frequency of intermediate monocytes (*r* = 0.8, *P* < 0.01) in PAs (Figure 8B). Such an association was absent in the LTNP cohort despite a similar pattern of apparent dysregulation in monocyte subsets and expansion of Tregs (Figure 8C). Monocyte-specific signatures of non-progression observed in this study have been summarized in Table 4.

**Table 4:** Immune signatures of non-progression.

	**Signatures unique to LTNP**	**Signatures similar to PA**	**Signatures similar to ART**
Absolute CD4 Count	Stable CD4 count in the absence of therapy		
Viral Load		High median viral load	
Monocyte subset frequency		Expansion of intermediate and decline of classical subsets	Expansion of intermediate and decline of classical subsets
Association of monocyte subset frequency with CD4 count and CD4/CD8 ratio			No correlation with CD4 counts and CD4/CD8 ratio
CD4 (MFI) expression on monocyte subsets	Significantly high CD4 on classical and intermediate subsets compared to SN		
HLA-DR (MFI) on monocyte subsets		Significantly high HLA-DR on intermediate subset compared to ART group	
Association of HLA-DR and CD4 (MFI)	Highly significant positive association on classical subset		Highly significant positive association on intermediate subset
CD206 frequency on monocytes subsets		Increase in CD206 expression on intermediate monocytes	
Association of CD206 frequency with viremia	Highly significant positive association of CD206 on all subsets with viremia		
T cell activation (frequency of CD4+ and CD8+ T cells)		Significantly high CD4+ and CD8+ T cell activation compared to SN	
Association of viremia with T cell activation	Significant association with CD4+ T cell activation		
Association of monocyte subset with Treg frequency	No association of monocyte subset and Tregs (unlike PAs)		

## Discussion

Several seminal studies have demonstrated the importance of soluble, serological markers of monocyte/macrophage activation in the pathogenesis of HIV infection as well as their prognostic value (17, 38–40). This study focussed on the dynamics of monocyte phenotypes and their implications for disease progression across disparate states including non-progression. The expansion of intermediate monocyte subset (CD14++CD16+) in HIV infection is associated with viral load and inversely with CD4 counts in ART-naïve individuals (8, 28). We report here for the first time that compared to seronegative controls HIV-1 infected long-term non-progressors in spite of sustained CD4 counts display a decline in frequency of classical monocytes and rise in intermediate monocytes similar to typical progressors. Interestingly, the LTNP group showed significant expansion of the non-classical subset as well. Non-classical monocytes or “patrolling” monocytes express abundant CX3CR1, fractalkine adhesion receptor, that enables trafficking to vascular endothelial surfaces under homeostatic conditions (41). In addition, as shown in murine models, they are recruited to sites of atherosclerotic plaque formation in a CCR5-dependent fashion (42). Intermediate monocytes, on the other hand, with their pro-inflammatory phenotype, independently predict adverse cardiovascular outcomes in HIV-uninfected individuals (43). An expansion of both pro-atherogenic monocyte subsets in non-progressors is reflective of chronic monocyte activation, potentially exposing this group to the risk of acquiring cardiovascular disease. Importantly, unlike what we observed in the PA group, the perturbation of monocytes subsets in non-progressors did not correlate with both CD4 counts and CD4/CD8 ratio. While, the lack of association with absolute CD4 counts could be due to clustering around a median value of ~500 cells/μL, an absence of association with CD4/CD8 ratio, despite the wide range, points to disparate mechanisms of immune homeostasis in LTNPs. A recent study from the French Hospital Database on HIV (ANRS C04) reported that LTNPs comprised of individuals with significant viremia and low CD4/CD8 ratio, as observed in our cohort (44). Low CD4/CD8 ratios were also independently associated with the risk of non-AIDS morbidity and mortality in virally suppressed individuals based on data from the CoRIS cohort (45). Thus, we speculate that expansion of activated monocyte subsets and low CD4/CD8 ratios could be driving factors for non-AIDS defining illnesses in our LTNP cohort.

Interestingly, we did not observe a significant difference in the frequency of both classical and intermediate subsets between treated (ART) and therapy-naïve (PA) participants. Partial restoration of the non-classical subset was observed. While our study does not reflect previous longitudinal observations (46), the ART group comprised of individuals receiving therapy for at least a year [median = 3.96 (1–10.25) years] and showed restoration of absolute CD4 counts. Our observations on persistence of monocyte subset dysregulation despite ART-mediated viral suppression and CD4 recovery are also supported by previous studies (28, 31, 32, 47) on the impact of HAART on the monocyte compartment.

Originally regarded as a transitional subset (between classical and non-classical), intermediate monocytes can be clearly distinguished based on certain unique markers such as CCR5 and HLA-DR (2). In our study, we observed the expression of HIV-binding receptors- CD4 and CCR5, to be highest on the intermediate subset across cohorts. We report for the first time that differential expression across subsets for CD4 is more prominent in ART-naïve groups irrespective of progression compared to treated participants with no significant difference between subsets in healthy individuals. Zhen et al. reported that CD4 (on monocytes) ligation with MHC-II molecule on activated endothelial cells triggers the differentiation of monocytes into functional macrophages (48). We also observed a rise in CD4 expression across monocyte subsets that was significantly higher on intermediate monocytes in LTNP and PA groups compared to seronegative controls. Upregulation of CD4 in viremic conditions coupled with inflamed vasculature known to be prevalent in HIV infection could result in increased differentiation of blood monocytes into macrophages at site of inflammation and contribute to plaque formation. Interestingly and in contrast, CCR5 expression on monocytes in LTNPs resembled seronegative controls. This may be a protective signature in LTNPs considering the recent encouraging results of CCR5 antagonist maraviroc (MVC) therapy in PLHIV that apparently demonstrate mitigation of exacerbatory CCR5 signaling (49–52).

Intermediate monocytes play a crucial role in antigen presentation and we report here an enhanced HLA-DR expression on this distinct subset in LTNPs similar to other infected cohorts (1, 2). Interestingly, non-progressors displayed high levels of monocyte activation in terms of elevated HLA-DR on all monocyte subsets comparable to typical progressors and higher than individuals on therapy. Contrary to our observations on dysregulation in frequencies of monocyte subsets that did not associate with typical markers of disease progression in LTNPs, the increase in HLA-DR on intermediate monocytes showed a positive association (not significant) with viral load and a stronger inverse association with CD4/CD8 ratio. It suggests that functional markers expressed on monocytes, but not dysregulation of subsets *per se*, may be predictive of immune status in non-progressors. In addition, ART was effective in partially restoring monocyte activation to the level of seronegative controls. Our data supplements what is already known regarding the suppressive effects of ART on HLA-DR expression (31, 32) and extends this data to LTNP individuals. We also report for the first time a significant association between per-cell expression of CD4 and HLA-DR on the intermediate subset in non-progressors and therapy receiving individuals. While in LTNPs, enhanced CD4 on monocytes could be attributed to ongoing viral replication and ensuing activation, the absence of a similar association in typical progressors leads us to speculate that the monocyte compartment may serve as an important site for infection in non-progressors thereby driving viremia. In individuals on ART, such an association could indicate residual viral replication in monocyte reservoirs.

Mannose receptor (CD206) is C-type lectin, identified as an alternate receptor for HIV-binding on monocyte derived macrophages (MDMs) by its ability to recognize the mannosylated residues of envelope protein gp120 (53, 54). CD206 is also a selective marker for alternatively-activated macrophages (M2 phenotype), especially those derived from blood monocytes (55). Our study is the first to report that mannose receptor expression is most prominent on the intermediate subset in seronegative controls and all HIV infected cohorts. HIV infected CD14+CD16+ monocytes preferentially transmigrate across the blood-brain barrier (BBB) seeding the CNS viral reservoir in HIV-associated neurocognitive disorders (HAND) (56). The presence of CD206, an alternate receptor for HIV-binding, on intermediate monocytes could increase the susceptibility of this subset.

Recent reports have highlighted the expression of CD206 on “M2”-like monocytes that populate circulation in disorders associated with autoimmunity and chronic inflammation (33–35, 57, 58). This prompted us to investigate the expression of CD206 across different states of HIV disease progression and we observed an increased expression of CD206 on intermediate monocytes in both PA and LTNP groups compared to treated participants. The frequency of CD206+ intermediate monocytes showed significant association with per-cell expression of HLA-DR on the same subset in seronegative controls and treated individuals. It is interesting to note that the disequilibrium between activation markers persisted irrespective of disease progression status (PA and LTNP) and was restored by ART. Li et al. reported that a higher frequency of CD206+ “M2”-like monocytes was accompanied by higher levels of plasma IL-10 in colorectal cancer and a similar observation was made by Hou et al. in *Helicobacter pylori* infection (33, 58). IL-10, an immunoregulatory cytokine elevated in untreated HIV infection but partially restored to normal levels by ART (59, 60), is produced by several cell types, most notably monocytes, whose IL-10 expression is in turn controlled by regulatory T cells (Tregs) (61). Activated Tregs can induce alternatively activated monocytes with upregulation of M2 specific marker CD206 and increased IL-10 production (62). As observed in our own dataset, both putative progressors and non-progressors showed higher frequency of Tregs (CD4+ CD25^high^ CD127^low^) compared to seronegative controls, that associated with the expansion of intermediate monocyte subset albeit only in typical progressors, not LTNP. Furthermore, IL-10 itself can polarize monocytes toward a deactivated “M2c” macrophage phenotype that expresses CD206 (63). We speculate that CD206 upregulation in ART-naïve groups could be IL-10 mediated and measuring these levels would be an important part of future studies. Intriguingly, the association of CD206 with viremia was highly significant in non-progressors, but not typical progressors, highlighting its utility as a surrogate for virological suppression in these individuals and further underscoring a unique monocyte-virus interplay that accompanies extended viral exposure in the presence of delayed CD4 depletion.

One of the limitations of this study was the heterogeneous nature of our LTNP cohort, which included both viremic non-progressors and individuals with low viremia. This presents the opportunity, in future studies; if possible, to recruit specifically viremic and non-viremic non-progressors to better delineate correlates of non-progression. Implementation of test-and-treat affected the availability of samples for longitudinal analysis in ART-naïve groups. Future study design would involve follow-up of some of these participants to examine the effects of delayed therapeutic intervention on immune restoration. Furthermore, we recognize a need to incorporate neopterin, sCD14, sCD163, and other soluble biomarkers associated with innate immune activation in our investigation. To the best of our knowledge this is the first study to examine dysregulation of the monocyte compartment in long-term non-progressors. It supports the use of immunomodulatory agents in addition to conventional anti-retroviral therapy to offset the potentially hazardous effects of continued immune activation in the presence of virological suppression.

## Data Availability Statement

The datasets generated for this study are available on request to the corresponding author.

## Ethics Statement

The studies involving human participants were reviewed and approved by ICMR-NIRRH Ethics Committee for Clinical Studies (Project No. 225/2012). Written informed consent to participate in this study was provided by the participants' legal guardian/next of kin.

## Author Contributions

VPat and VMP were involved in study design, data analysis, and writing the original manuscript. AS and VPad enrolled participants. VMP, AS, and VPad performed experiments. VN and PP enabled participant recruitment and collection of clinical history. VPat and VMP reviewed and edited the final manuscript.

### Conflict of Interest

The authors declare that the research was conducted in the absence of any commercial or financial relationships that could be construed as a potential conflict of interest.

## Abbreviations

SNAE: Serious non-AIDS events
HAART: highly active antiretroviral therapy
PA: Pre-ART
LTNP: long-term non-progressor
SN: seronegative controls
CVD: cardiovascular disease
HAND: HIV-associated neurocognitive disorders.
