# Supplementary material for: Monocyte Based Correlates of Immune Activation and Viremia in HIV-Infected Long-Term Non-Progressors
Source: Front Immunol. 2019 Dec 6;10:2849. doi: 10.3389/fimmu.2019.02849 (PMC6908494; doi:10.3389/fimmu.2019.02849)
Supplement: Supplementary file 1 [file Data_Sheet_1.PDF]

Supplementary Material

Supplementary Figures

Supplementary Figure 1:

Supplementary Figure 1: Clinical Characteristics of Participants

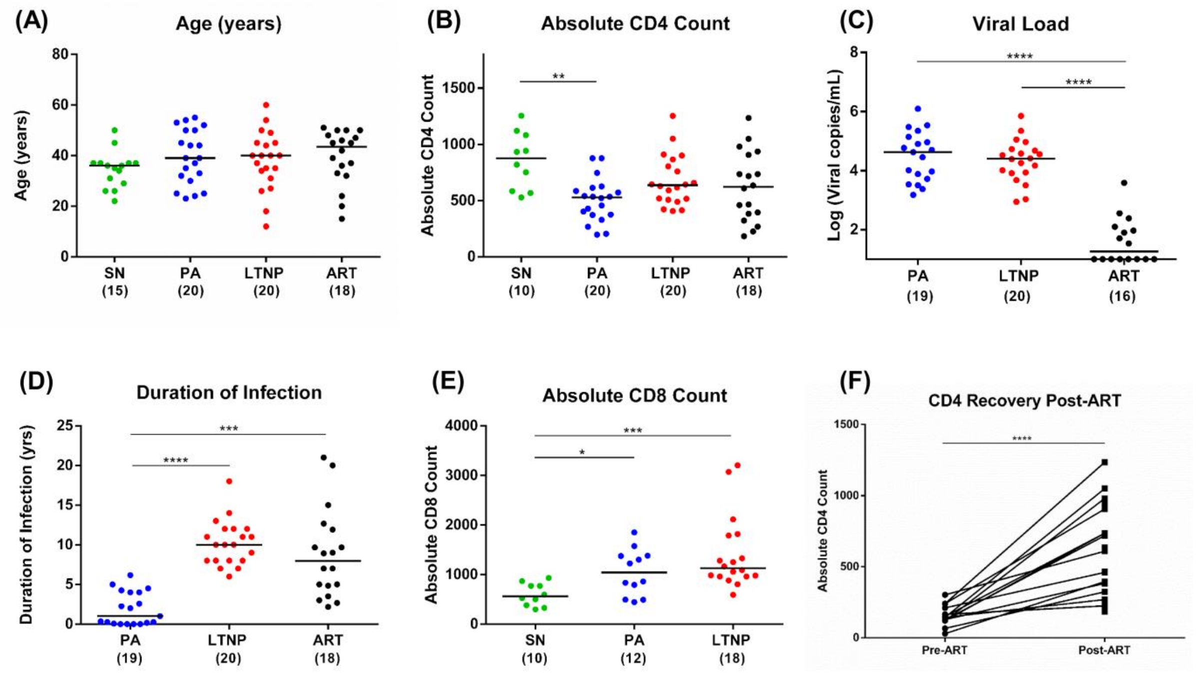

<u>Supplementary Figure 1: Clinical Characteristics of Participants</u> – A), B), C), D), & E) Scatter plots showing age, absolute CD4 count, viral load, duration of infection and absolute CD8 count of recruited participants respectively (horizontal bar indicates median). F) Before-after plot showing CD4 count recovery after initiation of antiretroviral therapy (n = 14). The two data points indicate the CD4 count immediately prior to initiation of therapy and at the time of sampling respectively. Duration on ART varies in different individuals as mentioned in Table 1. Data on CD4 nadir was not available for 4 participants. Statistical significance was estimated by Kruskal-Wallis ANOVA followed by Dunn's multiple comparison test; *, P<0.05; **, P<0.01; ***, P<0.001; ****, P<0.0001.

<u>Supplementary Figure 2:</u>

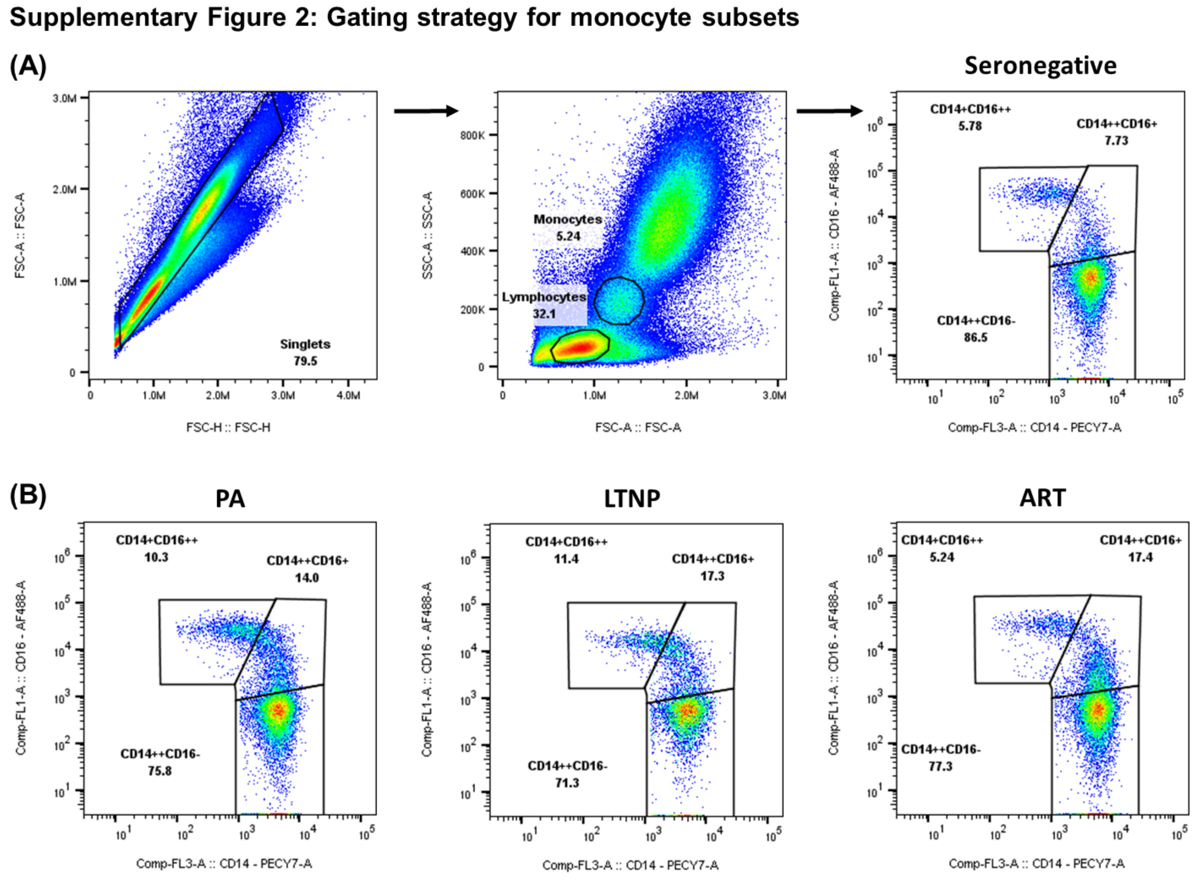

<u>Supplementary Figure 2: Gating strategy for monocyte subsets</u> - A) Gating strategy for delineation of monocyte subsets into classical (CD14++CD16-), intermediate (CD14++CD16+) and non-classical (CD14+CD16++) monocytes is shown in seronegative sample. B) Representative plots of subset distribution in three infected cohorts – PA, LTNP and ART groups respectively. *Participants in PA and LTNP groups had high viral load (>100,000 copies/mL) and absolute CD4 counts of 540 and 1051 cells/µL respectively. Participant on ART had undetectable viral load (<34 copies/mL) and CD4 count of 936 cells/µL.*

Supplementary Figure 3:

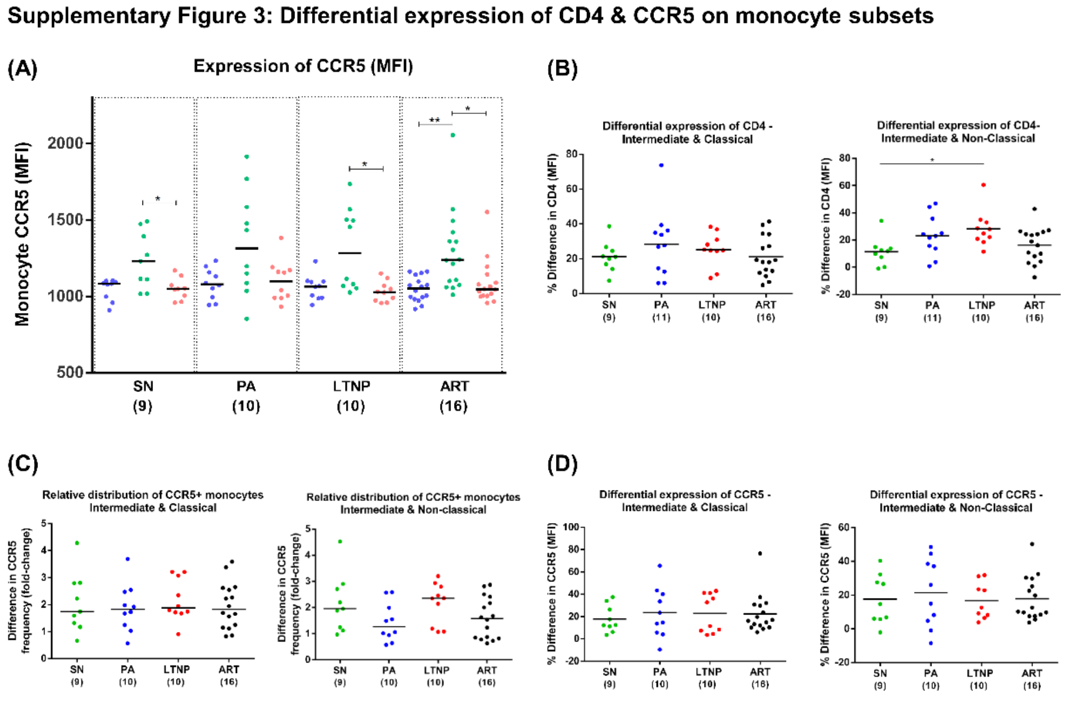

<u>**Supplementary Figure 3: Differential expression of CCR5 on monocyte subsets**</u> – A) Expression of HIV-binding co-receptor CCR5 (MFI) across monocyte subsets for each cohort. B) Differential expression was most pronounced in the ART-naïve groups where per –cell expression of CD4 was 23.05% [13.79 – 37.03%] and 26.40% [IQR – 20.3 – 33.44%] (P<0.05) higher on intermediate monocytes than non-classical monocytes in PAs and LTNPs respectively, compared to the 11.79% [IQR = 3.68 – 14.32%] increment observed in SN controls (right panel). CD4 per-cell expression was also 27.77% [IQR - 12.59 – 36.21%] and 24.99% [IQR – 21.06 – 32.08%] higher on intermediate monocytes than classical monocytes in PA and LTNP groups respectively but did not reach significance compared to SN controls [median = 21.02%, IQR = 15.45 – 25.58%] (left panel). C) Relative distribution of CCR5 (%) positivity between intermediate monocytes and classical (left panel) or non-classical (right panel) monocytes across cohorts. The fold-change in frequency of CCR5 positive cells between intermediate and non-classical subsets varied across groups with non-progressors [median = 2.35, IQR = 1.16 – 2.83] displaying a pattern of expression similar to SN individuals [median = 1.95, IQR = 1.18 – 2.8], whereas PAs [median = 1.275, IQR = 0.87 – 2.13] closely resembled individuals on therapy [median = 1.58, IQR = 0.83 – 2.35] D) Differential per-cell expression of CCR5 (MFI) between intermediate and classical (left panel) or non-classical (right panel) monocyte subsets across cohorts. The difference in CCR5 expression per-cell (MFI) between monocyte subsets was consistent across all cohorts. Kruskal-Wallis ANOVA followed by Dunn's multiple comparison test was used to assess significance; *, P<0.05; **, P<0.01; ***, P<0.001; ****, P<0.0001.

<u>Supplementary Figure 4:</u>

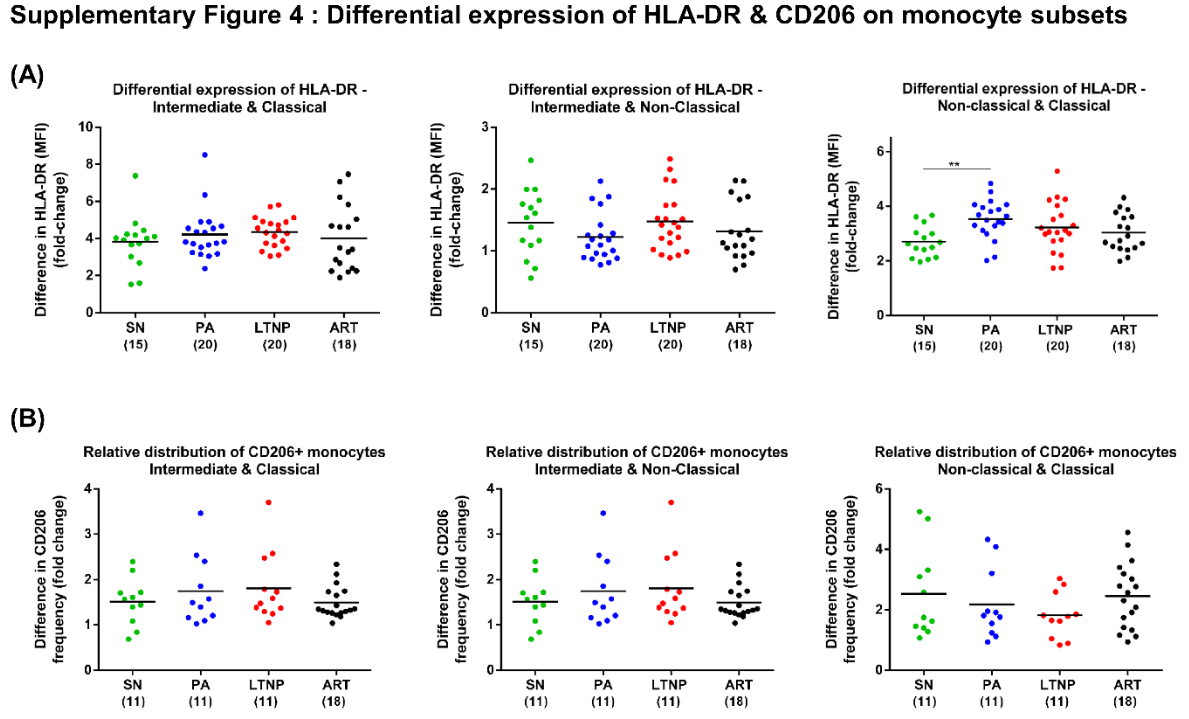

<u>Supplementary Figure 4: Differential expression of HLA-DR & CD206 on monocyte subsets</u> – A) & B) Differential per-cell expression of HLA-DR (MFI) & relative distribution of CD206 (%) positivity between monocyte subsets across cohorts. Kruskal-Wallis ANOVA followed by Dunn's multiple comparison test was used to assess significance; *, P<0.05; **, P<0.01; ***, P<0.001; ****, P<0.0001.

Supplementary Figure 5:

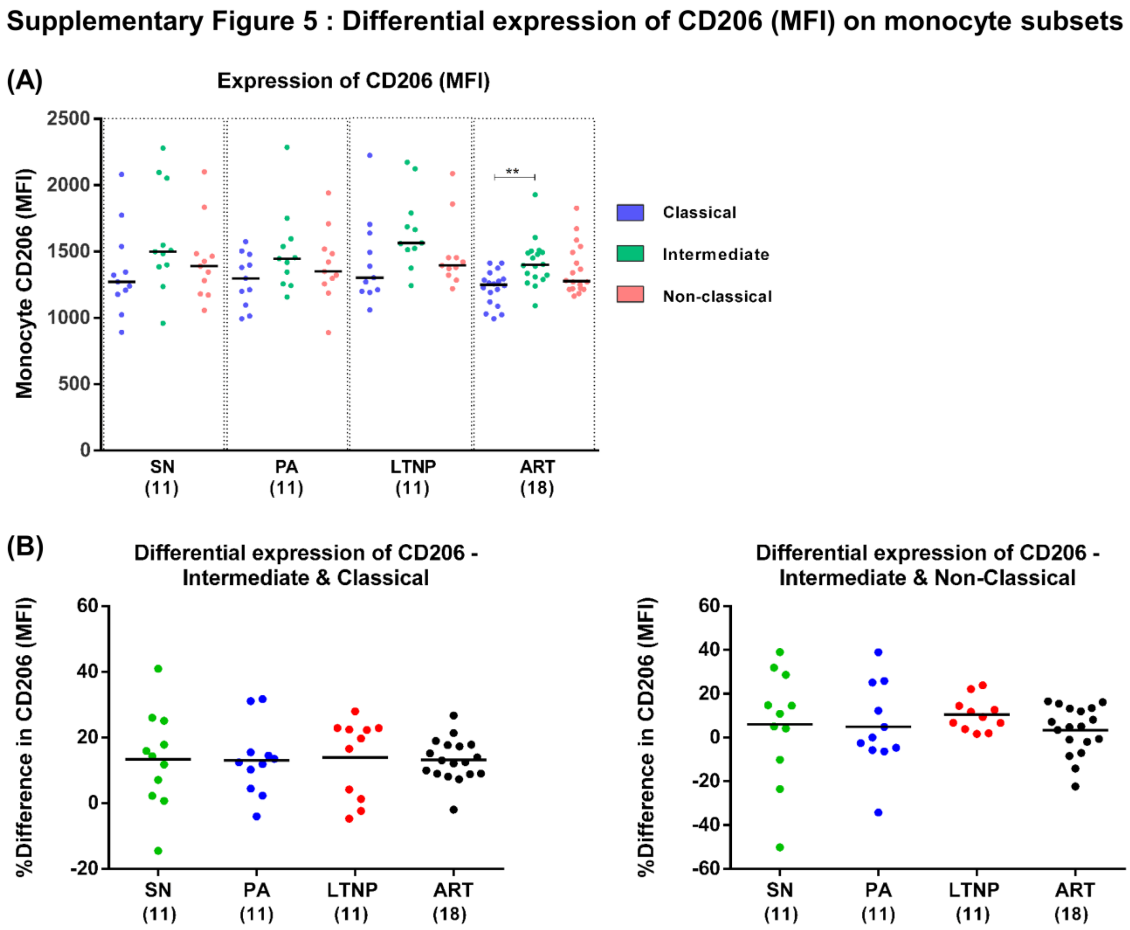

Supplementary Figure 5: A) Expression of M2 phenotype marker CD206 (MFI) across monocyte subsets for each cohort. B) Differential per-cell expression of CD206 (MFI) between intermediate and classical (left panel) or non-classical (right panel) monocyte subsets across cohorts. The difference in CD206 per-cell expression (MFI) between monocyte subsets was consistent across all cohorts. Kruskal-Wallis ANOVA followed by Dunn's multiple comparison test was used to assess significance; *, $P<0.05$; **, $P<0.01$; ***, $P<0.001$; ****, $P<0.0001$.

Supplementary Figure 6:

Supplementary Figure 6: Expression of CCR5 on monocyte subsets across cohorts

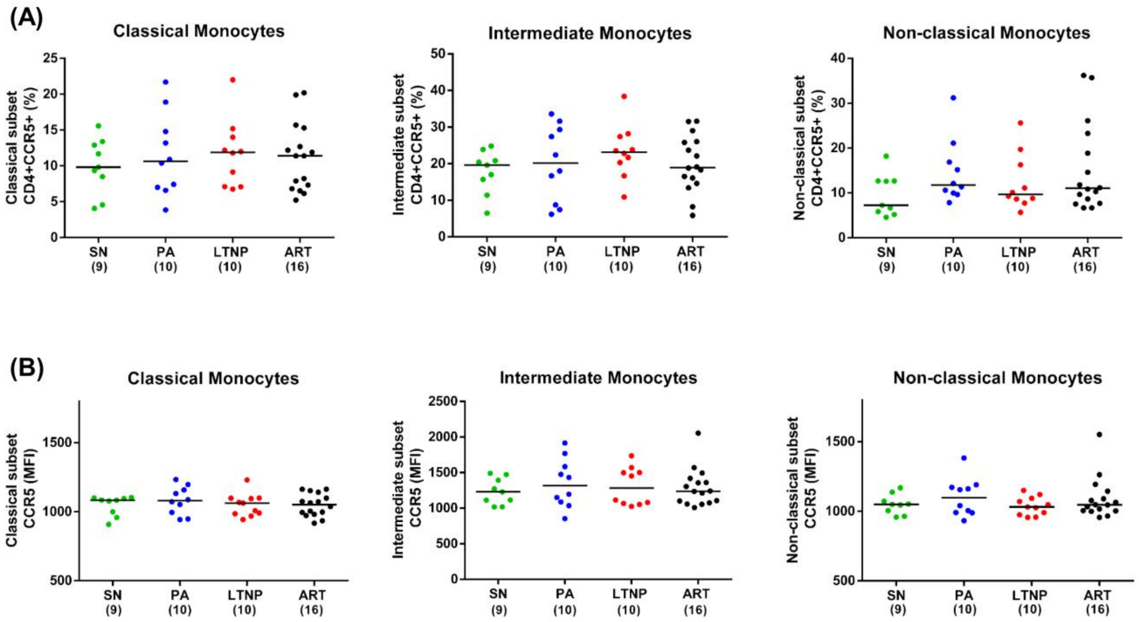

Supplementary Figure 6: Expression of CCR5 on monocyte subsets across cohorts- A) & B) Expression of CCR5 in terms of frequency and median fluorescence intensity respectively across cohorts.

Supplementary Figure 7:

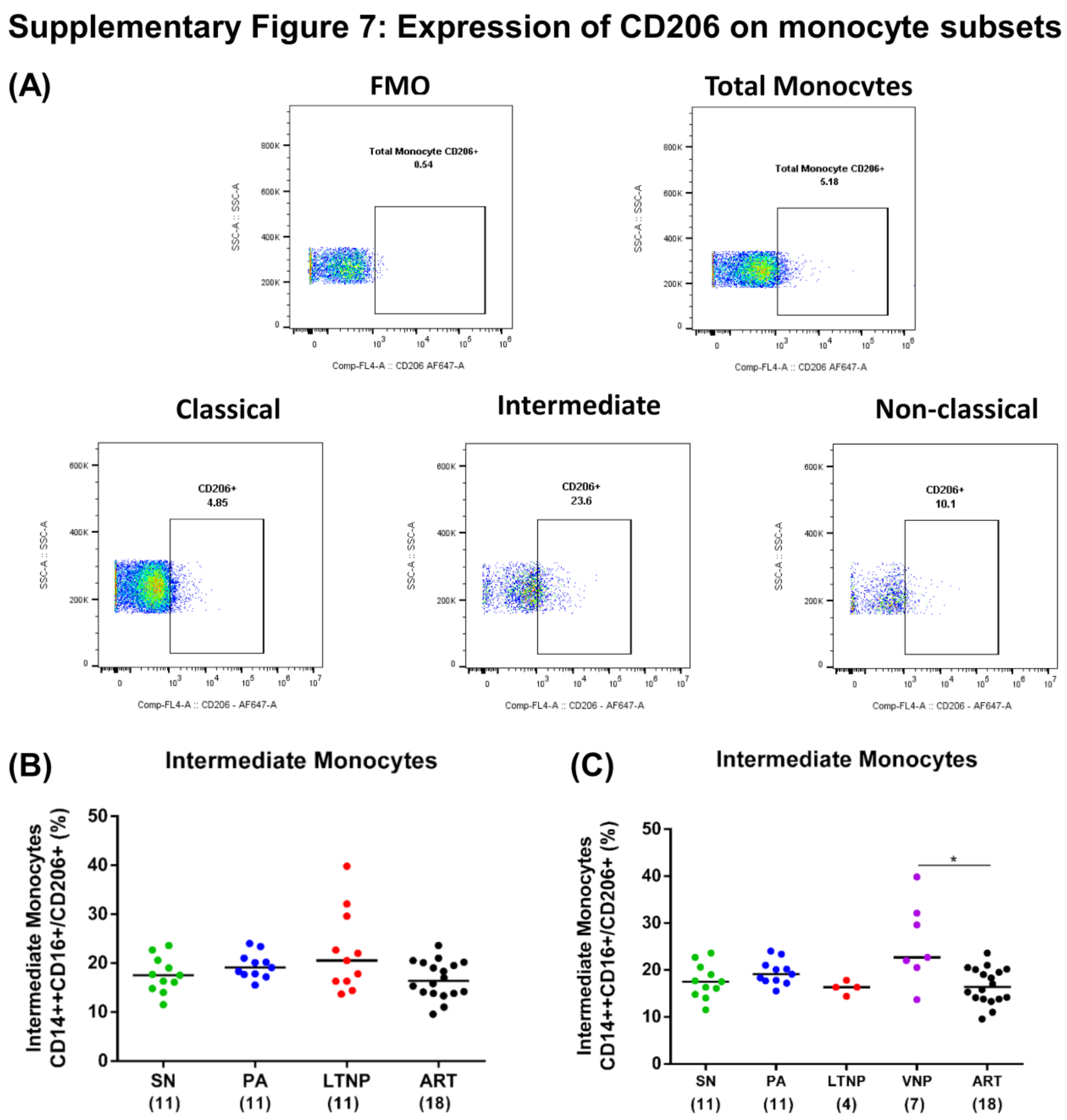

Supplementary Figure 7: Expression of CD206 on monocyte subsets - A) Gating strategy for CD206 on classical, intermediate and non-classical monocytes based on FMO controls. B) CD206 expression on intermediate monocytes in PA, LTNP, ART and SN groups. C) Non-progressors were stratified based on viral load into individuals with low viremia (LTNP) (VL<10,000 copies/mL) and relatively high viremia - viremic non-progressors (VNPs) (VL>10,000 copies/mL) and CD206 expression was compared across all relevant groups – PA, LTNP, VNP, ART and SN groups. Kruskal-Wallis ANOVA followed by Dunn’s multiple comparison test was used to assess significance; *, $P<0.05$; **, $P<0.01$; ***, $P<0.001$; ****, $P<0.0001$. Although not significant by ANOVA, CD206 expression on intermediate monocytes in VNPs was observed to be significantly higher than SN, LTNP & ART groups by pairwise comparisons using unpaired t test ($P<0.05$ for all).

<u>Supplementary Table 1: Association of absolute CD4 count with CCR5 per-cell expression on monocyte subsets</u>

Group	N	Classical	Intermediate	Non-classical
PA	10	P = *0.0656** r = -0.5818	P = *0.0708** r = -0.5727	P = 0.2788 r = -0.3818
LTNP	11	P = 0.5135 r = -0.2364	P = 0.7330 r = -0.1273	P = 0.4697 r = -0.2606
ART	16	P = **0.0104** r = -0.6284	P = **0.0153** r = -0.6029	P = 0.1618 r = -0.3676

* Strong association nearing significance. **Bold** font if P value is significant.

<u>Supplementary Table 2: Association of HLADR per-cell expression with CD4 counts, CD4/CD8 ratio & viral load in therapy-naïve groups</u>

	PA			LTNP		
	Classical	Intermediate	Non-classical	Classical	Intermediate	Non-classical
Log (Viral Load)	**P = 0.0517*** **r = 0.4526**	P = 0.5471 r = 0.1474	P = 0.3180 r = 0.2421	P = 0.9448 r = -0.0165	P = 0.1224 r = 0.3569	P = 0.1401 r = 0.3419
Absolute CD4 Count	P = 0.9699 r = -0.009	P = 0.9098 r = 0.0271	P = 0.4998 r = 0.1602	**P = 0.0823 *** **r = -0.3979**	P = 0.1582 r = -0.3278	P = 0.7001 r = -0.0918
CD4/CD8 Ratio	P = 0.8871 r = 0.0501	P = 0.2947 r = -0.3417	P = 0.4380 r = -0.2551	P = 0.4252 r = -0.2004	**P = 0.0563*** **r = -0.4574**	P = 0.7975 r = -0.0651

* Strong association nearing significance.
